# Predicting long-term clinical mortality of elderly patients with vertebral compression fractures

**DOI:** 10.3389/fmed.2026.1708134

**Published:** 2026-04-21

**Authors:** Shuofan Wang, Kaiwen Peng, Kaili Peng, Zhichao Gao

**Affiliations:** 1 Department of Orthopedics, The First People’s Hospital of Linping District, Hangzhou, Zhejiang, China; 2 Department of Radiology, Liqun Hospital, Shanghai, China; 3 Department of Gastroenterology, The First People’s Hospital of Linping District, Hangzhou, Zhejiang, China

**Keywords:** elderly, machine learning, mortality, SHAP (shapley additive explanations), vertebral compression fractures (VCFs)

## Abstract

Vertebral compression fractures (VCFs) are prevalent among the elderly, often leading to significant complications and mortality. We aimed to develop and validate a predictive model for long-term mortality in patients with VCFs, utilizing a comprehensive dataset from a single-center retrospective study. A total of 440 patients aged 65 years and older, diagnosed with VCFs between 2017 and 2020, were included. The participants were divided into a training set (*n* = 296) and a validation set (*n* = 144). We employed five survival analysis models: Cox Proportional Hazards, LASSO, Random Survival Forests, Gradient Boosting Machine, and Extreme Gradient Boosting (XGB). The XGB model demonstrated superior predictive performance, achieving a C-index of 0.753 with the top five predictive variables, outperforming other models in the validation set. SHAP analysis revealed age, sex, previous fracture, history of cancer, and co-morbidity as significant predictors of mortality. The model’s robustness was confirmed through Kaplan–Meier survival analysis, which showed significant stratification of high- and low-risk groups (*p* < 0.001). Calibration curves and decision curve analysis further validated the model’s clinical utility. The XGB model’s interpretability was enhanced using SHAP values, providing insights into the influence of individual features on mortality risk. In conclusion, we developed a novel risk-stratification model for predicting mortality for VCFs. Our model can also aid in stratifying patients, with high discriminative ability. The use of an explainable machine learning model can aid physicians in making individualized treatment decision in VCFs patients.

## Introduction

Vertebral compression fractures (VCFs) are a common, highly disabling injury, especially among elderly patients (1). Approximately 1.7 million vertebral compression fractures occur in America and in Europe annually, and osteoporosis remains the most common cause of vertebral compression fractures (2). VCFs may cause prolonged back pain, spinal deformity due to vertebral body compression, functional disability, decreased quality of life, adjacent vertebral fractures, and increased short- and long-term mortality (3). Traditional treatment modalities, including conservative management and surgical interventions, have demonstrated variable efficacy in this demographic, primarily due to the presence of comorbidities and diminishing physiological reserves (4). To date, however, there is a lack of standard predictive models to predict prognosis for elderly patients with VCFs for clinicians. Consequently, there is an urgent need for enhanced prognostic tools that can accurately predict patient outcomes and inform clinical decision-making.

Recently, a number of comprehensive studies have uncovered that various clinical, radiological, and comorbid factors may significantly influence the prognosis of elderly patients suffering from VCFs (5). Previous research has clearly demonstrated noteworthy correlations between several variables, including age, sex, body mass index (BMI), anemia, and the presence of comorbid conditions such as chronic obstructive pulmonary disease (COPD) and diabetes, all of which play a crucial role in determining overall survival rates in patients with VCFs (6–10). This realization highlights the multitude of influencing factors that can impact patient outcomes, emphasizing the importance of understanding these variables in clinical practice. Consequently, the integration of these risk factors to develop a robust prognostic model becomes particularly vital in improving patient care. One promising approach to achieve this is through the application of machine learning (ML), a powerful technique that facilitates automatic feature extraction and is increasingly recognized as a key component in the realization of advanced artificial intelligence algorithms (11, 12).

In the present study, we aimed to establish a ML-based model based on regularly utilized and easily accessible clinical variables for predicting survival probabilities in patients aged 65 and older who suffer from thoracolumbar VCFs. By building this model, it may aid in stratifying patients, with high discriminative ability. The use of an explainable ML model can aid physicians in making individualized treatment decision in VCFs patients.

## Methods

### Data source

The datasets utilized in this study are accessible via Zenodo,^1^ an open-access repository that offers a wide array of discoverable and freely reusable reference research data. The authors have relinquished their copyright claims to these original research datasets.

### Patients

A secondary analysis was conducted utilizing data from a single-center, retrospective study (13). This study encompassed patients aged 65 years and older who were diagnosed with acute VCFs in the thoracic or lumbar regions, specifically at levels T5-L5, during the period from January 1, 2017, to December 31, 2020. The study exclusively included patients without without underlying oncological process and those who underwent either conservative treatment or surgical interventions limited to vertebral augmentation procedures. The inclusion period was determined based on the availability of departmental records, ensuring that all patients had a minimum follow-up duration of 2 years, which was crucial for assessing survival outcomes. Patients with incomplete follow-up data (less than 2 years) or those requiring vertebral arthrodesis due to insufficient fracture healing were excluded from the study. Furthermore, only the initial occurrence of a VCFs was considered for patients who experienced a subsequent fracture within the first 3 months.

### Data collection

We systematically collected data encompassing demographic characteristics, as well as epidemiological, clinical, diagnostic, and therapeutic variables. Initially, we employed manual feature selection to identify potential variables, guided by clinical expertise, existing literature, and data accessibility. Variables with less than 10% missing data were retained for further analysis (14). Among these retained variables, the overall rate of missing data was 1.16%, and missing values were imputed using the k-nearest neighbor algorithm (15). Multicategory nominal variables, such as sex, were processed using One-Hot encoding (16). Ultimately, our structured database comprised 13 clinical variables, referred to as “features,” for candidate predictors. Notable predictive indicators included age, stay in hospital (days), sex, previous fracture, history of cancer, chronic steroid treatment, traumatic VCFs, osteoporosis, located at the thoracic, multiple fractures, treatment, co-morbidity hospitalization, and outpatient geriatric care. Additionally, we determined the vital survival status and follow-up duration for each patient with VCFs.

### Model training

For the purpose of model development and validation, the dataset was divided into a 70% training set and a 30% test set. The baseline characteristics of each group are presented in Supplementary Table S1. The Kaplan–Meier (KM) curve analysis indicated no statistically significant difference in survival probabilities between the training and validation sets following random allocation (*p* = 0.660) (Supplementary Figure S1). To improve the stratification strategy and ensure that the training and test sets exhibited similar distributions, including both event and time-to-event variables, a modified stratified data-splitting approach was implemented. We employed five survival analysis methods: Cox Proportional Hazards (CoxPH), LASSO, Random Survival Forests (RSF), Gradient Boosting Machine (GBM), and Extreme Gradient Boosting (XGB). The rationale for employing this diverse set of models was to comprehensively compare different modeling strategies: CoxPH as the traditional parametric benchmark; LASSO for feature selection and regularization; and RSF, GBM, and XGB as advanced tree-based ensemble methods known for handling complex, non-linear relationships. This comparative approach ensures the selected model demonstrates robust performance on our dataset. Model performance was evaluated and compared using the Harrell’s concordance index (C-index) on the validation set. The final model selection was based on its superior predictive accuracy (highest C-index) and stability. Each of these methods has been previously adapted into survival models capable of accommodating right-censored survival data (17).

The survival models generate a probability that an event will occur by a specific time, such as the likelihood of survival within a five-year period in our study. A higher score suggests a greater probability of the event, in this case, survival, occurring earlier within the specified timeframe. The predictive performance of the models was evaluated using the Harrell concordance index (c-index), a metric particularly suited for assessing predicted risk scores. The models were trained and optimized using the training dataset. Hyperparameter optimization was conducted using the “Optuna” framework, employing a repeated 5-fold cross-validation strategy aimed at maximizing the c-index. A total of 100 trials were performed, and for each model, the set of parameters that achieved the highest c-index was selected. Subsequently, the test dataset was employed to evaluate and compare the performance of the models.

### Model explanation

SHAP (shapley additive explanations) is a technique for model interpretability that is grounded in game theory principles (18). It quantifies the contribution of individual feature values to a model’s prediction by assigning an explanatory score to each feature. The SHAP values are derived from the concept of Shapley values, which involve permuting and integrating feature values to evaluate their influence on the model’s output. SHAP analysis facilitates the understanding of the underlying factors driving model predictions, offering intuitive explanations for the predicted outcomes. This approach not only enhances comprehension of the model’s decision-making logic and aids in identifying key risk factors but also provides a scientific basis for developing individualized therapeutic strategies. Although accurately interpreting machine learning models remains a challenge, the SHAP method offers a robust solution by ranking input features and elucidating prediction outcomes, thereby addressing the “black-box” problem.

### Statistical analysis

Continuous variables were evaluated for normality through the application of the Shapiro–Wilk test. Variables exhibiting normal distribution were analyzed using the student’s t-test for comparisons between two groups. In contrast, variables that did not conform to a normal distribution were subjected to the Kruskal-Wallis test. Categorical variables were assessed using Pearson’s chi-squared test, or Fisher’s exact test in cases of small cell counts. The Kaplan–Meier method was utilized to generate survival curves, which were subsequently compared using the multivariate log-rank test. All statistical analyses were conducted using R version 4.5.0, employing the mlr3verse, mlr3proba, mlr3extralearners, tidyverse, survival, compareGroups, and gtsummary (version 2.0.4) packages. Statistical significance was determined at a threshold of *p* < 0.05, with a two-sided criterion.

## Results

### Patient characteristics

A total of 440 eligible VCFs patients were ultimately included in the study, with the training set included 296 patients, and the validation set included 144 patients. Among the 440 patients, 144 patients (32.7%) died during the follow-up period. Table 1 summarizes the baseline characteristics of the overall cohort and provides a comparison based on the outcome. The results showed that patients who died were significantly older (median age 84.0 vs. 76.0 years, *p* < 0.001), more likely to be male (41.7% vs. 20.3%, *p* < 0.001), had a higher prevalence of previous oncological disease diagnosis (34.0% vs. 15.9%, *p* < 0.001), were less likely to have a traumatic fracture mechanism (51.4% vs. 66.2%, *p* = 0.004), and had a higher rate of comorbidities (morbidity: 32.6% vs. 17.6%, *p* = 0.001). While not statistically significant after adjustment, deceased patients also showed trends towards higher rates of previous fractures (36.8% vs. 28.4%, *p* = 0.093) and less frequent comprehensive geriatric outpatient care (12.5% vs. 7.1%, *p* = 0.09). No significant differences were found for hospital length of stay, chronic steroid use, previous osteoporosis diagnosis, fracture location (thoracic), fracture multiplicity (single/multiple), or treatment type (brace vs. vertebral augmentation).

**Table 1:** Baseline characteristics of patients in the alive and dead patients.

Baseline characteristics	All patients (*N* = 440)	Alive (*N* = 296)	Dead (*N* = 144)	*p* value
Age, Median	79 (73–84)	76 (71–81)	84 (77–88)	<0.001
Stay in hospital (days), Median	4 (2–10)	4 (2–9)	5 (2–10)	0.489
Follow time (days), Median	1,270 (870–1722)	1,475 (1150–1820)	669 (294–1,037)	<0.001
Sex, *N* (%)				<0.001
Male	120 (27.273%)	60 (20.270%)	60 (41.667%)	
Female	320 (72.727%)	236 (79.730%)	84 (58.333%)	
Previous fracture, *N* (%)				0.093
No	303 (68.864%)	212 (71.622%)	91 (63.194%)	
Yes	137 (31.136%)	84 (28.378%)	53 (36.806%)	
History of cancer, *N* (%)				<0.001
No	344 (78.182%)	249 (84.122%)	95 (65.972%)	
Yes	96 (21.818%)	47 (15.878%)	49 (34.028%)	
Chronic steroid treatment, *N* (%)				0.389
No	383 (87.045%)	261 (88.176%)	122 (84.722%)	
Yes	57 (12.955%)	35 (11.824%)	22 (15.278%)	
Traumatic VCFs, *N* (%)				0.004
No	170 (38.636%)	100 (33.784%)	70 (48.611%)	
Yes	270 (61.364%)	196 (66.216%)	74 (51.389%)	
Osteoporosis, diagnosis, *N* (%)				0.162
No	262 (59.545%)	169 (57.095%)	93 (64.583%)	
Yes	178 (40.455%)	127 (42.905%)	51 (35.417%)	
Located at the thoracic, *N* (%)				1
No	217 (49.318%)	146 (49.324%)	71 (49.306%)	
Yes	223 (50.682%)	150 (50.676%)	73 (50.694%)	
Multiple fractures, *N* (%)				0.363
No	337 (76.591%)	231 (78.041%)	106 (73.611%)	
Yes	103 (23.409%)	65 (21.959%)	38 (26.389%)	
Treatment, *N* (%)				0.415
Brace	378 (85.909%)	251 (84.797%)	127 (88.194%)	
Vertebral augmentation	62 (14.091%)	45 (15.203%)	17 (11.806%)	
Co-morbidity hospitalization, *N* (%)				0.001
No	341 (77.500%)	244 (82.432%)	97 (67.361%)	
Yes	99 (22.500%)	52 (17.568%)	47 (32.639%)	
Outpatient geriatric care, *N* (%)				0.09
No	401 (91.136%)	275 (92.905%)	126 (87.500%)	
Yes	39 (8.864%)	21 (7.095%)	18 (12.500%)	

### Model development

Comparison of five survival models using the C-index revealed XGB as the optimal predictor of long-term mortality (Table 2). When trained with all the 13 variables, XGB achieved the highest validation set performance (C-index = 0.748), marginally exceeding RSF (0.739), GBM (0.738), and CoxPH (0.738). Crucially, using only the top 5 predictive variables (age, sex, previous fracture, history of cancer, and co-morbidity hospitalization), XGB uniquely improved its validation performance to 0.753 - the highest observed result across all models and configurations. In contrast, all other models exhibited reduced test performance with feature reduction: RSF declined to 0.717 (from 0.739), GBM to 0.721 (from 0.738), CoxPH to 0.715 (from 0.738), and Lasso improved modestly to 0.711 (from 0.683) but remained lower than XGB. XGB demonstrated exceptional robustness to feature reduction, gaining +0.005 in validation C-index, while competitors showed declines of 0.018–0.023. This superior absolute performance with both feature sets and unique stability established XGB as the optimal model. Supplementary Figure S2 showed the hyperparameter space of the models.

**Table 2:** Comparing the predictive performance of different models.

C-index	CoxPH	Lasso	RSF	GBM	XGB
Models trained with all variables					
Train set	0.782	0.726	0.825	0.803	0.817
Test set	0.738	0.683	0.739	0.738	0.748
Models trained with top 5 variables					
Train set	0.746	0.703	0.820	0.797	0.826
Test set	0.715	0.711	0.717	0.721	0.753

### Model interpretation

**Figure 1:**
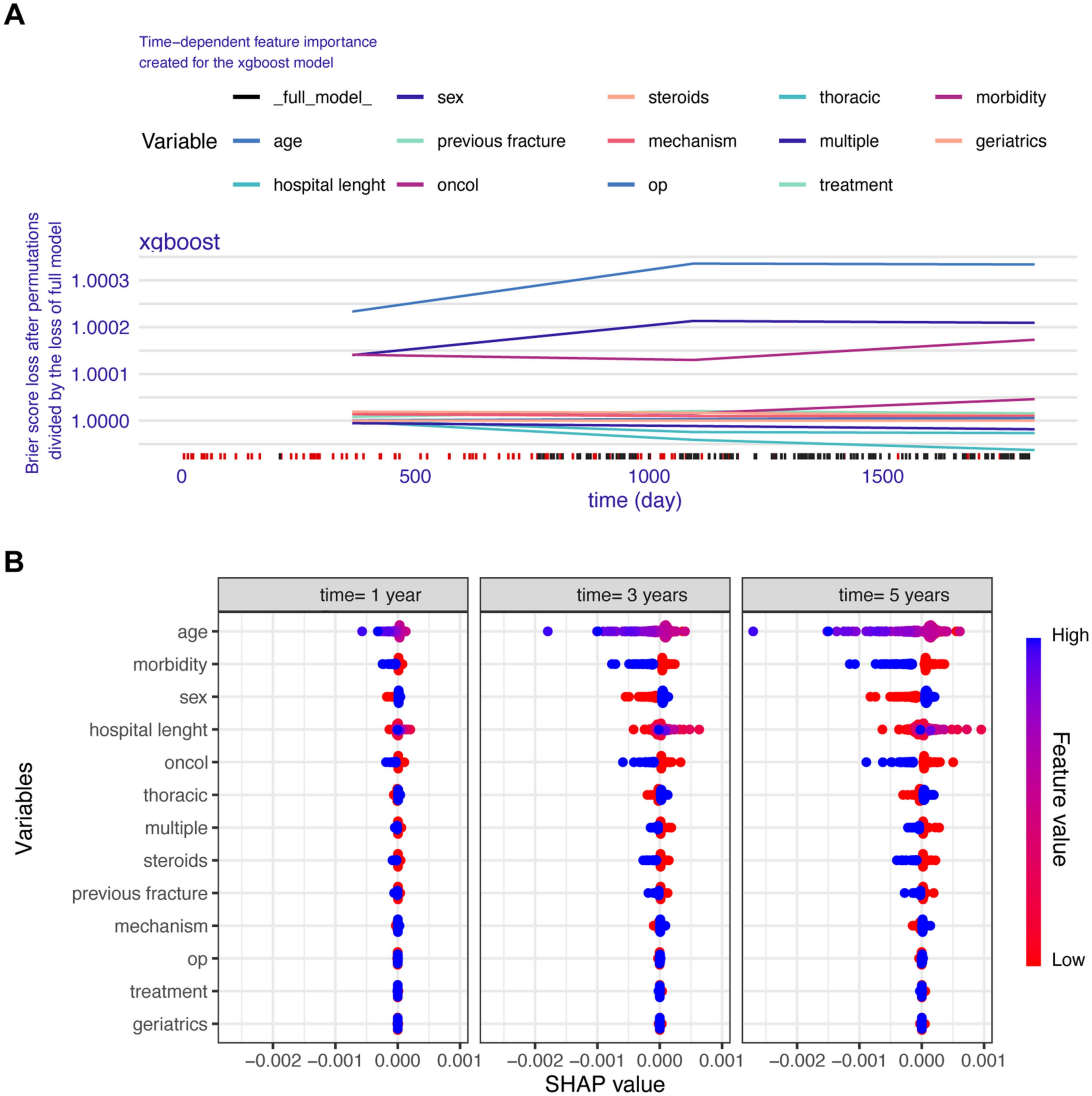
The global interpretation of the XGB model and feature importance. **(A)** The time-dependent feature importance of the XGB model by using the Brier score loss after permutations divided by the loss of full model. **(B)** The global interpretability of the XGB model, along with the 13 influential features, is shown in the SHAP summary plot.

The time-dependent feature importance of the XGB model revealing the importance of variables influencing the mortality in VCFs patients (Figure 1A). Feature importance trajectories show age persistently dominates mortality prediction across all timepoints, with morbidity consistently ranking second. Oncol (cancer history) exhibits progressive importance escalation after year 1, while hospital length peaks acutely then decline. The global interpretability of the XGB model, along with the 13 inuential features, is shown in the SHAP summary plot (Figure 1B). SHAP values at 1/3/5 years confirm consistent risk directionality: advanced age, higher morbidity, male sex, cancer presence, and prolonged hospitalization increase mortality risk.

**Figure 2:**
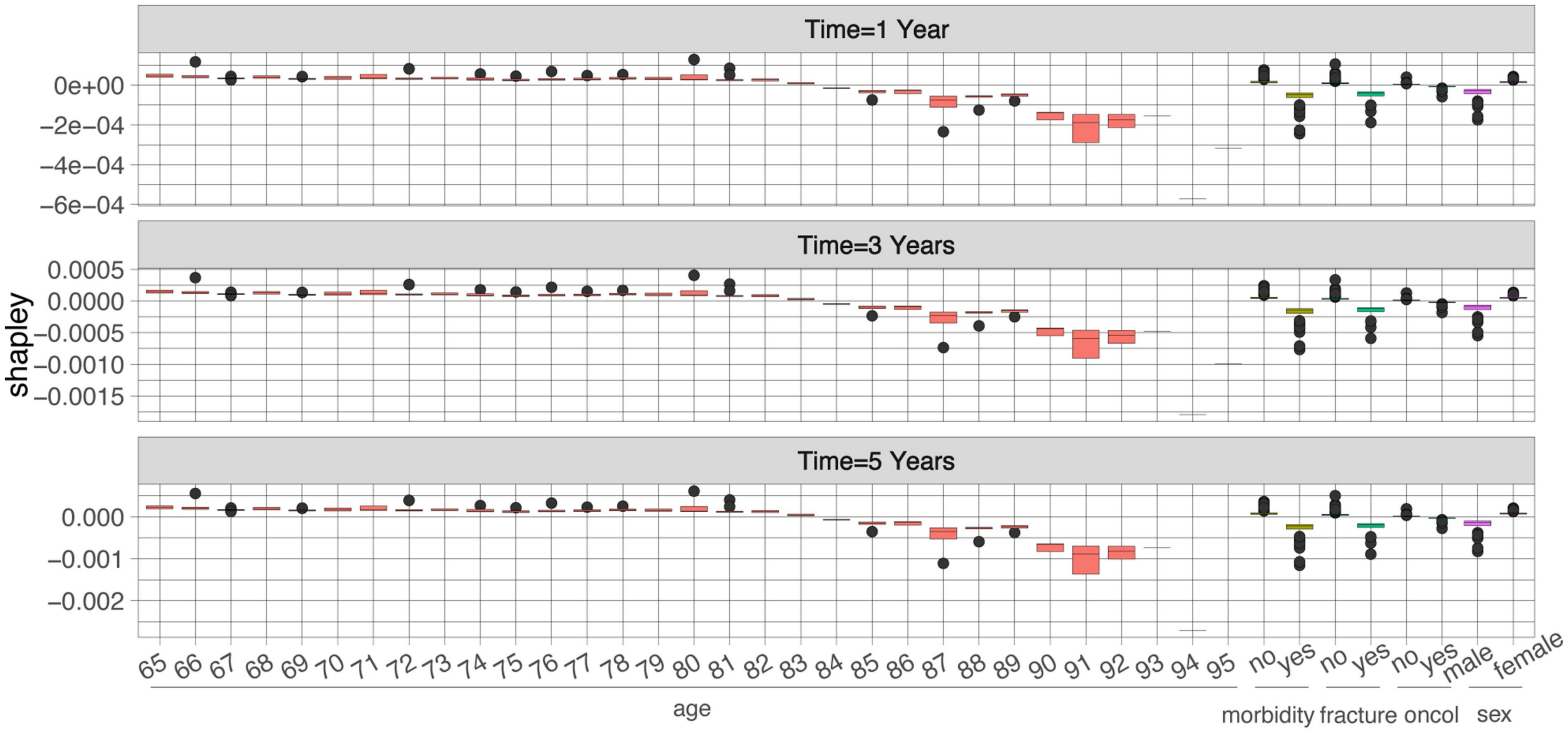
SHAP feature dependence plots. The SHAP dependence plot shows how a single feature affects the output of the prediction model, as seen with age, co-morbidity, previous fracture, history of cancer, and sex. SHA*p* values for specific features exceed zero, representing an increased likelihood of clinical remission occurrence. The *x*-axis represents the feature value, and the *y*-axis represents the feature attribution to the predicted result. Each data point corresponds to 1 prediction from a particular patient.

Figure 2 presents SHAP summary plots identifying the top five drivers of predicted mortality risk at 1, 3, and 5 years post-vertebral fracture in XGB model. Age was the dominant predictor across all time horizons, with higher age consistently increasing risk (positive SHAP values). Comorbidity burden ranked highly, where greater burden elevated predicted mortality. Fracture characteristics exerted their strongest influence on 1-year risk. The presence of an oncological history significantly increased predicted risk, becoming increasingly impactful for 3- and 5-year mortality. Male sex was uniformly associated with modestly higher risk estimates compared to female sex. Critically, the direction of influence for all top features aligned with clinical expectations: advanced age, higher comorbidity, more severe fracture characteristics, active/historical cancer, and male sex all contributed positively to predicted mortality risk.

### Model performance

**Figure 3:**
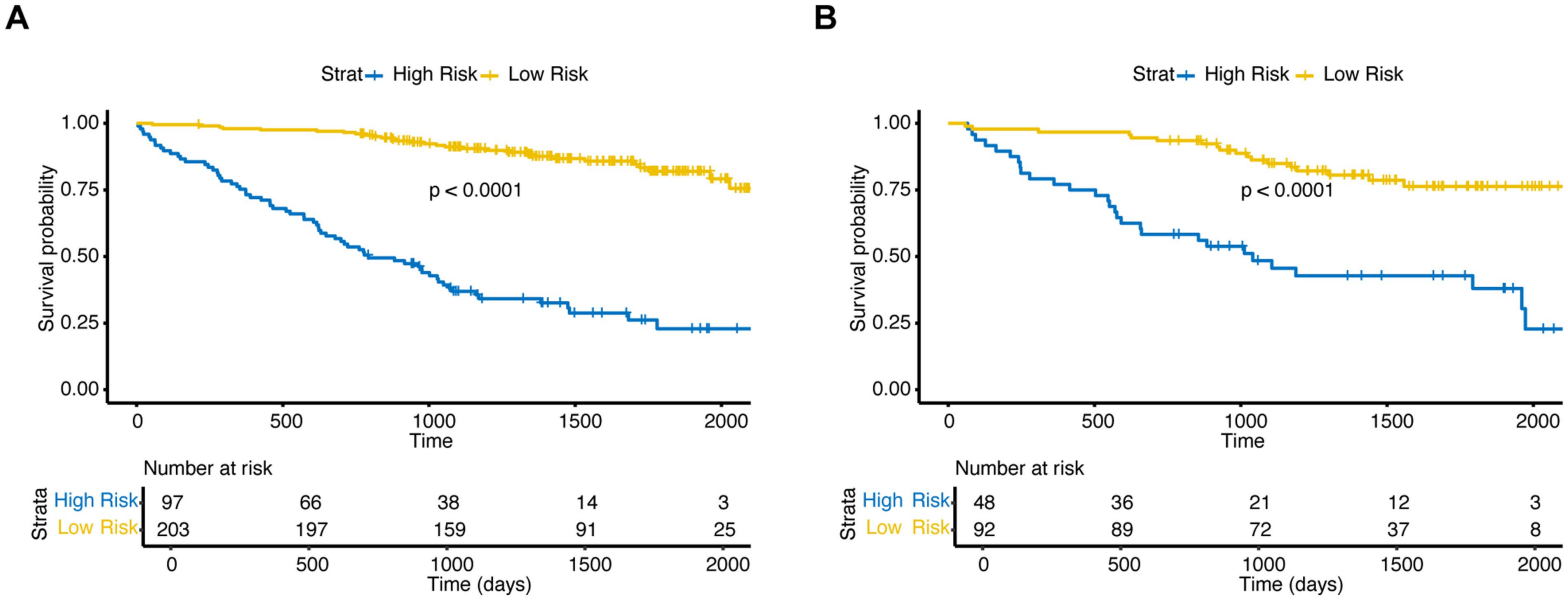
XGB model (trained with top 5 variables) discrimination in the validation set. **(A)** Kaplan–Meier curves according to the predicted risk scores in the training set. **(B)** Kaplan–Meier curves according to the predicted risk scores in the validation set.

The model showed good performance in risk stratification, demonstrating a proportional decrease in alive status with increased risk scores. In the training set, Kaplan–Meier curves revealed significantly lower survival probability in the high-risk group versus low-risk group (*p* < 0.001, Figure 3A). This discriminative ability was validated in the validation set (*p* < 0.001, Figure 3B), where the high-risk group showed accelerated mortality versus the low-risk group. The model consistently identified patients with substantially poorer long-term prognoses.

**Figure 4:**
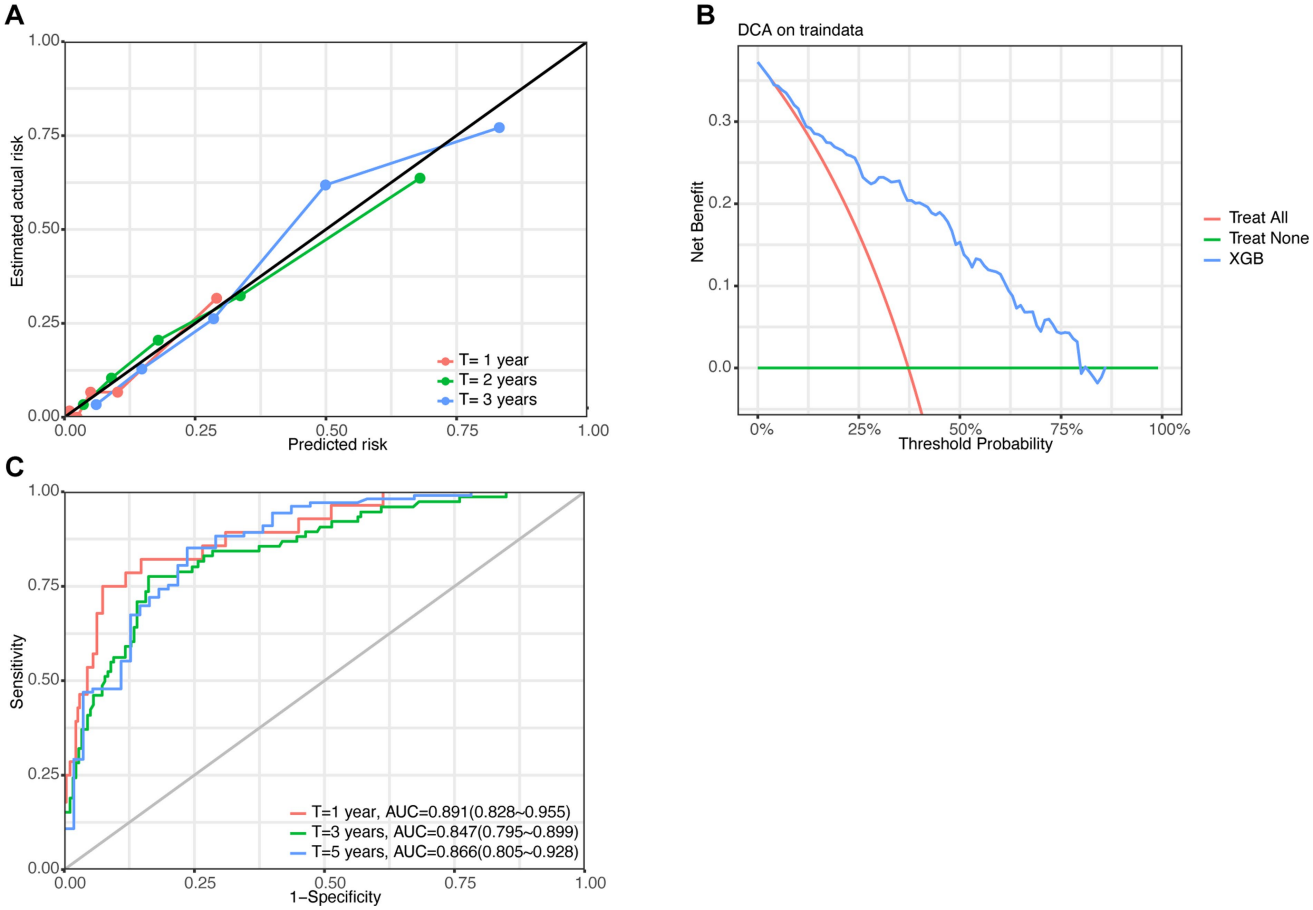
XGB model (trained with top 5 variables) performance in the validation set. **(A)** Calibration curves show good agreement between predicted and actual mortality at 1/3/5 years. **(B)** Decision curve analysis confirms superior clinical utility of the XGB model over threshold probabilities 0–75%. **(C)** Time-dependent AUC values indicate moderate to good discrimination.

We also evaluated XGB model performance in the training set. Calibration curves at 1/2/3 years show excellent alignment between predicted and observed mortality (points closely tracking the diagonal) (Figure 4A). Decision curve analysis demonstrates superior clinical utility of the model (blue curve) versus “treat-all” and “treat-none” strategies across risk thresholds (Figure 4B). ROC curves reveal outstanding discrimination: AUC = 0.891 (95%CI: 0.828–0.955) at 1 year, 0.847 (0.795–0.899) at 3 years, and 0.866 (0.805–0.928) at 5 years, confirming robust time-dependent predictive accuracy (Figure 4C). The XGB model performance in the validation set was shown in Supplementary Figure S3.

## Discussion

Thoracolumbar VCFs significantly impact the elderly due to osteoporosis, falls, and comorbidities, leading to pain, disability, and higher mortality (19). Patients with different risk levels had different prognoses related to different clinical factors. Accordingly, there is an urgent need for better prognostication models for elderly patients with VCFs in this setting. In the present study, we used a retrospective clinical and demographic data and established a reliable prognostic XGB-based ML model to predict survival probabilities in elderly patients with VCFs. Key predictors include age, sex, previous fracture, history of cancer, and co-morbidity hospitalization. Our model might improve the prognostic accuracy and risk stratification, aiding in better clinical management for elderly VCFs.

The predictive validity of the model was evaluated by partitioning the raw dataset into training and validation subsets to assess its performance. KM analysis revealed significant differences in overall survival outcomes between the high-risk and low-risk cohorts. Within the training set, the AUC values for predicting survival at 1, 3, and 5 years were 0.891, 0.847, and 0.866, respectively. In contrast, the validation set yielded slightly lower AUC values of 0.803 for 1 year, 0.793 for 3 years, and 0.633 for 5 years. To further evaluate the model’s efficacy, calibration curve analysis and DCA were conducted, both of which demonstrated that the model possessed good calibration and clinical utility.

Numerous studies have examined the risk factors influencing the prognosis of patients with VCFs. Zhang et al. demonstrated that adherence to the mediterranean diet exerts a moderating effect on the relationship between VCFs patients and mortality (20). Chu et al. identified that morbid obesity is linked to an elevated risk of adverse short-term outcomes in patients undergoing vertebroplasty or kyphoplasty for osteoporotic vertebral compression fractures, including unfavorable discharge and specific complications (21). Furthermore, Zhang et al. reported that vertebral augmentation significantly reduces the mortality risk associated with osteoporotic VCFs, especially in the early stages post-fracture, with kyphoplasty proving more reliable and effective than vertebroplasty in mitigating mortality risk (22). Conversely, Gold et al. found no apparent mortality benefit from kyphoplasty among patients with vertebral fractures (23). These findings underscore the complexity and diversity of factors affecting VCFs prognosis. Currently, predictive models for VCFs prognosis are limited, and our research aims to address this knowledge gap.

The research methods employed in this study exhibit several strengths that enhance the robustness and reliability of the findings. Firstly, the study utilizes a large and well-defined cohort of 440 patients, specifically focusing on individuals aged 65 and older, which improves the generalizability of the results to the population most affected by VCFs. The rigorous inclusion and exclusion criteria, along with a minimum follow-up duration of 2 years, ensure that the assessment of survival outcomes is accurate and reflective of real clinical scenarios. Moreover, the integration of ML techniques, particularly the XGB model, effectively manages complex, high-dimensional data, capturing intricate patterns and interactions among various clinical, demographic, and comorbidity factors that traditional statistical methods might miss. The use of SHAP values for model interpretation addresses the “black-box” nature of ML algorithms and provides clinically relevant insights into the key predictors of mortality risk, which can facilitate individualized patient management strategies. Additionally, comprehensive statistical analyses, including the Harrell concordance index and KM survival curves, further validate the predictive accuracy and clinical utility of the developed model. Together, these methodological strengths highlight the potential of this research to significantly improve prognostic capabilities and inform treatment decisions for elderly patients with VCFs.

The present study has several limitations that warrant consideration. Firstly, the retrospective design inherently restricts the ability to establish causality between the identified predictors and mortality outcomes. While the study utilized a comprehensive dataset, the reliance on historical records may introduce biases related to data completeness and accuracy, particularly concerning comorbidities and treatment interventions. Additionally, the study’s single-center nature may limit the generalizability of the findings to broader populations, as variations in clinical practices and patient demographics across different healthcare settings could influence outcomes. Furthermore, while ML models, particularly XGB, demonstrated robust predictive performance, the interpretability of such models remains a challenge, and the reliance on SHAP values, although beneficial, may not fully capture the complexities of clinical decision-making. Lastly, the study’s focus on a limited set of clinical variables may overlook other potentially relevant factors, such as psychosocial elements or patient preferences, which could further refine prognostic assessments. Future research should aim to validate these findings in diverse cohorts and explore the integration of additional variables to enhance the predictive accuracy and clinical applicability of survival models in this patient population.

The predictive model and SHAP analysis provide actionable insights for clinical practice. The identification of the top five predictors (age, sex, previous fracture, history of cancer, and co-morbidity hospitalization) offers a straightforward framework for risk stratification. Clinicians can use this model to identify high-risk patients (e.g., older males with a history of cancer or multiple comorbidities) who may benefit from more intensive management strategies, including comprehensive geriatric assessment, optimized comorbidity control, fall prevention programs, and closer follow-up. The significant association of a cancer history with long-term mortality underscores the need for integrated oncology and geriatric care in this population. For future research, external validation in multi-center, prospective cohorts is essential to confirm generalizability. The next steps should also focus on translating the model into a practical clinical decision support tool, potentially integrated within electronic health records, to facilitate point-of-care risk assessment. Furthermore, incorporating additional variables such as imaging biomarkers, genetic data, or social determinants of health could enhance predictive accuracy and pave the way for more personalized intervention strategies.

In conclusion, our study identified and validated a XGB model to predicting long-term clinical mortality of elderly patients with VCFs. Our model can also aid in stratifying patients, with high discriminative ability. The use of an explainable machine learning model can aid physicians in making individualized treatment decision in VCFs patients.

## Data Availability

All the data used in this study were obtained from the Zenodo Digital Repository database (https://zenodo.org/records/7738365).

## Footnotes

1
https://zenodo.org/records/7738365
