# Supplementary material for: Predicting long-term clinical mortality of elderly patients with vertebral compression fractures
Source: Front Med (Lausanne). 2026 Apr 21;13:1708134. doi: 10.3389/fmed.2026.1708134 (PMC13138989; doi:10.3389/fmed.2026.1708134)
Supplement: Supplementary file 1 [file Table_1.docx]

| **Table S1. Baseline demographics and clinical characteristics of patients in the training set and validation set.** | | | | |
| --- | --- | --- | --- | --- |
|  | All patients  (N=440) | Training set  (N=300) | Validation set (N=140) | P value |
| Age, Median | 79 (73-84) | 79 (72-84) | 78 (74-85) | 0.64 |
| Stay in hospital (days), Median | 4 (2-10) | 5 (2-10) | 4 (2-9) | 0.428 |
| sex, N (%) |  |  |  | 0.282 |
| male | 120 (27.273%) | 87 (29.000%) | 33 (23.571%) |  |
| female | 320 (72.727%) | 213 (71.000%) | 107 (76.429%) |  |
| Previous fracture, N (%) |  |  |  | 0.52 |
| no | 303 (68.864%) | 210 (70.000%) | 93 (66.429%) |  |
| yes | 137 (31.136%) | 90 (30.000%) | 47 (33.571%) |  |
| History of cancer, N (%) |  |  |  | 0.991 |
| no | 344 (78.182%) | 234 (78.000%) | 110 (78.571%) |  |
| yes | 96 (21.818%) | 66 (22.000%) | 30 (21.429%) |  |
| Chronic steroid treatment, N (%) |  |  |  | 0.678 |
| no | 383 (87.045%) | 263 (87.667%) | 120 (85.714%) |  |
| yes | 57 (12.955%) | 37 (12.333%) | 20 (14.286%) |  |
| Traumatic VCFs, N (%) |  |  |  | 0.178 |
| no | 170 (38.636%) | 109 (36.333%) | 61 (43.571%) |  |
| yes | 270 (61.364%) | 191 (63.667%) | 79 (56.429%) |  |
| Osteoporosis, diagnosis, N (%) |  |  |  | 0.977 |
| no | 262 (59.545%) | 178 (59.333%) | 84 (60.000%) |  |
| yes | 178 (40.455%) | 122 (40.667%) | 56 (40.000%) |  |
| Located at the thoracic, N (%) |  |  |  | 0.766 |
| no | 217 (49.318%) | 146 (48.667%) | 71 (50.714%) |  |
| yes | 223 (50.682%) | 154 (51.333%) | 69 (49.286%) |  |
| Multiple fractures, N (%) |  |  |  | 0.947 |
| no | 337 (76.591%) | 229 (76.333%) | 108 (77.143%) |  |
| yes | 103 (23.409%) | 71 (23.667%) | 32 (22.857%) |  |
| Treatment, N (%) |  |  |  | 0.002 |
| brace | 378 (85.909%) | 269 (89.667%) | 109 (77.857%) |  |
| vertebral augmentation | 62 (14.091%) | 31 (10.333%) | 31 (22.143%) |  |
| Co-morbidity hospitalization, N (%) |  |  |  | 0.327 |
| no | 341 (77.500%) | 228 (76.000%) | 113 (80.714%) |  |
| yes | 99 (22.500%) | 72 (24.000%) | 27 (19.286%) |  |
| Outpatient geriatric care, N (%) |  |  |  | 0.159 |
| no | 401 (91.136%) | 269 (89.667%) | 132 (94.286%) |  |
| yes | 39 (8.864%) | 31 (10.333%) | 8 (5.714%) |  |
| Outcome, N (%) |  |  |  | 0.714 |
| alive | 296 (67.273%) | 204 (68.000%) | 92 (65.714%) |  |
| dead | 144 (32.727%) | 96 (32.000%) | 48 (34.286%) |  |
| Follow time (days), Median | 1270 (870-1722) | 1270 (870-1727) | 1268(877-1688) | 0.892 |

**
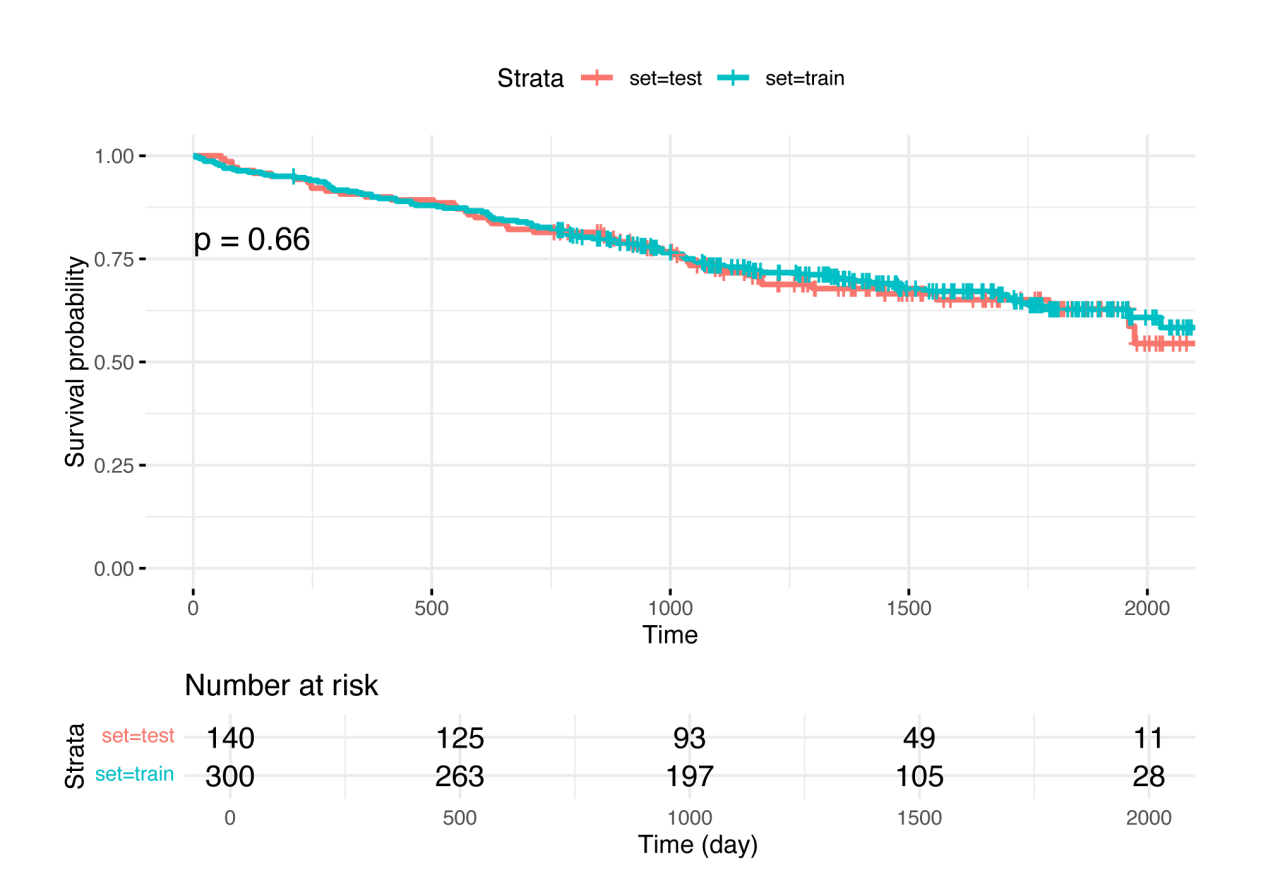
**

**Figure S1.** Kaplan-Meier curve between training set (n=300) and test set (n=140).

**
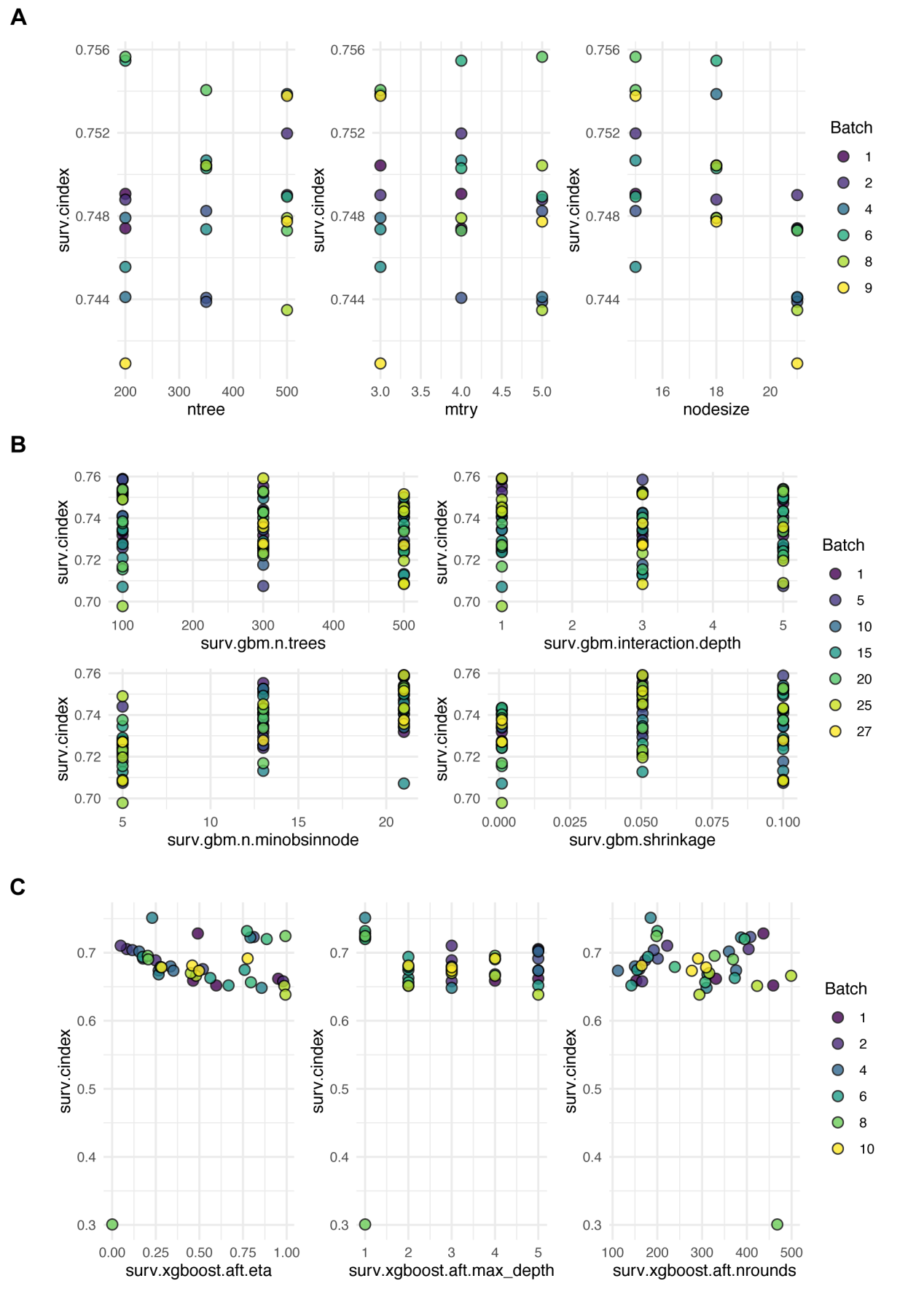
**

**Figure S2. Hyperparameter space of the models.** (A)The optimal RSF model utilized 200 trees (ntree), considered 5 variables per split (mtry), and employed a minimum node size of 15 (nodesize). This configuration achieved a cross-validated C-index of 0.756 during tuning. When trained on the full dataset, the model demonstrated strong discriminative ability with a training C-index of 0.825 for mortality risk stratification in vertebral fracture patients. The survival forest incorporated 13 predictors, with logrank splitting rules applied through resampling (swor) of 190 cases per tree. (B)The GBM was optimized with 300 trees, interaction depth=1, minimum node size=21, and learning rate=0.0505. Eleven of thirteen predictors contributed meaningfully to mortality prediction. The model achieved a training C-index of 0.803, demonstrating strong discriminative ability for stratifying vertebral fracture patients' mortality risk. These hyperparameters balanced model complexity and performance, with 300 iterations providing optimal predictive accuracy without overfitting the training cohort. (C) The XGBoost model was optimized with a learning rate (eta) of 0.229, tree depth of 1, and 185 boosting rounds. It achieved a training C-index of 0.817 and cross-validated tuning performance of 0.751. Using the AFT survival objective, this configuration demonstrated robust discriminative ability for predicting long-term mortality in vertebral fracture patients.


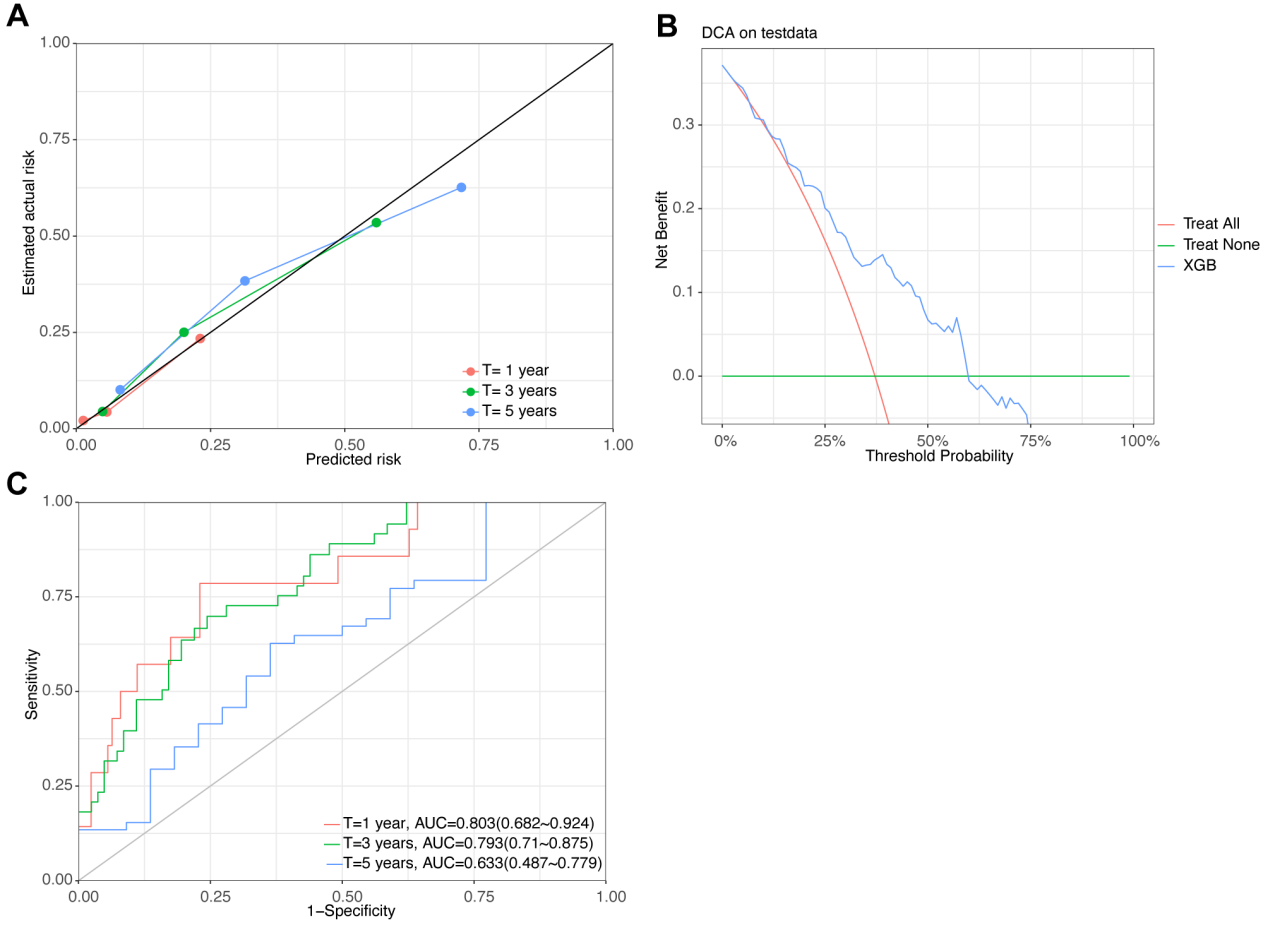


**Figure S3. XGB model performance in the test set.** (A) Calibration curves show good agreement between predicted and actual mortality at 1/3/5 years. (B) Decision curve analysis confirms superior clinical utility of the XGB model over threshold probabilities 0-75%. (C) Time-dependent AUC values indicate moderate to good discrimination (1-year: 0.803, 95%CI:0.682–0.924; 3-year: 0.793, 0.710–0.875; 5-year: 0.633, 0.487–0.779).
